# The efficacy of a suppository based on Phenolmicin P3 and Bosexil (Mictalase®) in control of irritative symptoms in patients undergoing thulium laser enucleation of prostate: a single-center, randomized, controlled, open label, phase III study

**DOI:** 10.1186/s12894-022-00974-0

**Published:** 2022-02-12

**Authors:** Riccardo Bertolo, Chiara Cipriani, Matteo Vittori, Marco Carilli, Francesco Maiorino, Valerio Iacovelli, Carlo Ganini, Michele Antonucci, Marta Signoretti, Filomena Petta, Massimo Panei, Pierluigi Bove

**Affiliations:** 1 Department of Urology, “San Carlo di Nancy” Hospital – GVM Care and Research, Via Aurelia 275, 00165 Rome, Italy; 2 Urology Unit, Department of Surgery, Tor Vergata University of Rome, Rome, Italy; 3 Torvergata Oncoscience Research TOR, Department of Experimental Medicine, University of Rome Tor Vergata, Rome, Italy; 4 Urology Unit, Department of Life, Health and Environmental Sciences, University of L’Aquila, Coppito, AQ Italy

**Keywords:** Benign prostatic hyperplasia, BPH, Laser enucleation, ThuLEP, LUTS, Urinary tract infections

## Abstract

**Background:**

Several studies described post-operative irritative symptoms after laser enucleation of prostate, sometimes associated with urge incontinence, probably linked to laser-induced prostatic capsule irritation, and potential for lower urinary tract infections We aimed to evaluate the efficacy of a suppository based on Phenolmicin P3 and Bosexil (Mictalase®) in control of irritative symptoms in patients undergoing thulium laser enucleation of prostate (ThuLEP).

**Methods:**

In this single-center, prospective, randomized, open label, phase-III study, patients with indication to ThuLEP were enrolled (Dec2019–Feb2021—Institutional ethics committee STS CE Lazio approval no.1/N-726—ClinicalTrials.gov NCT05130918). The report conformed to CONSORT 2010 guidelines. Eligible patients were 1:1 randomized. Randomization defined Group A: patients who were administered Mictalase® suppositories twice a day for 5 days, then once a day for other 10 days; Group B: patients who did not receive Mictalase® (“controls”). Study endpoints were evaluated at 15 and 30 days postoperation. Primary endpoint included evaluation of effects of the suppository on irritative symptoms by administering IPSS + QoL questionnaire. Secondary endpoint included evaluation of effects on urinary tract infections by performance of urinalysis with urine culture.

**Results:**

111 patients were randomized: 56 in Group A received Mictalase®. Baseline and perioperative data were comparable. At 15-days, no significant differences were found in terms of IPSS + QoL scores and urinalysis parameters. A significant difference in the rate of positive urine cultures favored Group A (*p* = 0.04). At 30-days follow-up, significant differences were found in median IPSS score (6 [IQR 3–11] versus 10 [5–13], Group A vs B, respectively, *p* = 0.02). Urinalysis parameters and rate of positive urine cultures were not significantly different.

**Conclusions:**

The present randomized trial investigated the efficacy of Mictalase® in control of irritative symptoms and prevention of lower urinary tract infections in patients undergoing ThuLEP. IPSS improvement 30-days postoperation was more pronounced in patients who received Mictalase®. Lower rate of positive urine culture favored Mictalase® group 15-days postoperatively.

***Trial registration*:**

The clinical trial has been registered on ClinicalTrials.gov on November 23rd, 2021—Registration number NCT05130918.

## Introduction

Lower urinary tract symptoms (LUTS) secondary to benign prostatic hyperplasia (BPH) are among the most common medical issues for the aging male [1]. Indeed, population-based studies have suggested that more than 40% of men aged over 60 years old suffers from BPH symptoms [2].

In patients bothered from LUTS caused by benign prostatic obstruction (BPO) refractory to medical therapy and worthy of surgical intervention, transurethral resection of the prostate (TURP) and open prostatectomy (OP) have been the reference-standard procedures [3]. Although effective, such treatment options are burdened by several potential perioperative morbidities. Over the past three decades, research focused on the development of new surgical strategies aimed to reduce procedure-related morbidity and complications. Thanks to the advent of laser technologies, endoscopic enucleation of the prostate (EEP) techniques have been developed [4, 5]. They reproduce the concept of OP, but this is achieved endoscopically like TURP, using a laser instead of a finger to enucleate the adenoma. In this scenario, the thulium laser enucleation of prostate (ThuLEP) has been introduced in 2010 [6] and represents a viable option suggested by the European Association of Urology (EAU) guidelines [7] starting from > 30 ml BPH. It has been reported that ThuLEP would either de-obstruct and reduce morbidity, catheterization time and hospital stay compared to TURP and OP [8–11]. Nevertheless, several studies described post-operative irritative symptoms after laser enucleation of prostate, sometimes associated with urge incontinence, probably linked to laser-induced prostatic capsule irritation, and potential for lower urinary tract infections [12]. A recent meta-analysis of eight studies calculated a pooled incidence of such symptoms around 9% amongst patients who underwent enucleation of the prostate for BPH. These symptoms negatively impact on patients’ quality of life, and their management is controversial. As such, in most of the available studies, no specific treatment was reported. In this scenario, the use of oral medical treatments and suppositories has been described with variable effectiveness [12–15]: analgesics and non-steroidal anti-inflammatory drugs to reduce the intensity of the discomfort; prednisone or betamethasone as an alternative; specific urinary tract analgesics such as phenazopyridine to manage dysuria; opioids as a debated option to control pain; gabapentin or pregabalin to relief pain via their neuropathic properties; finally, anticholinergic drugs and β_3_-agonists as an option when pain and/or dysuria are associated with urgency [12].

To contribute to this field, the present study was conceived to evaluate the efficacy of a suppository based on Phenolmicin P3 and Bosexil (Mictalase®) in the control of irritative symptoms and prevention of lower urinary tract infections in patients who underwent ThuLEP.

Both compounds have reported to have anti-inflammatory, antioxidant and anti-microbial properties in a variety of inflammatory diseases whose physio-pathological pathways are shared with those of prostatitis.

## Materials and methods

**Fig. 1:**
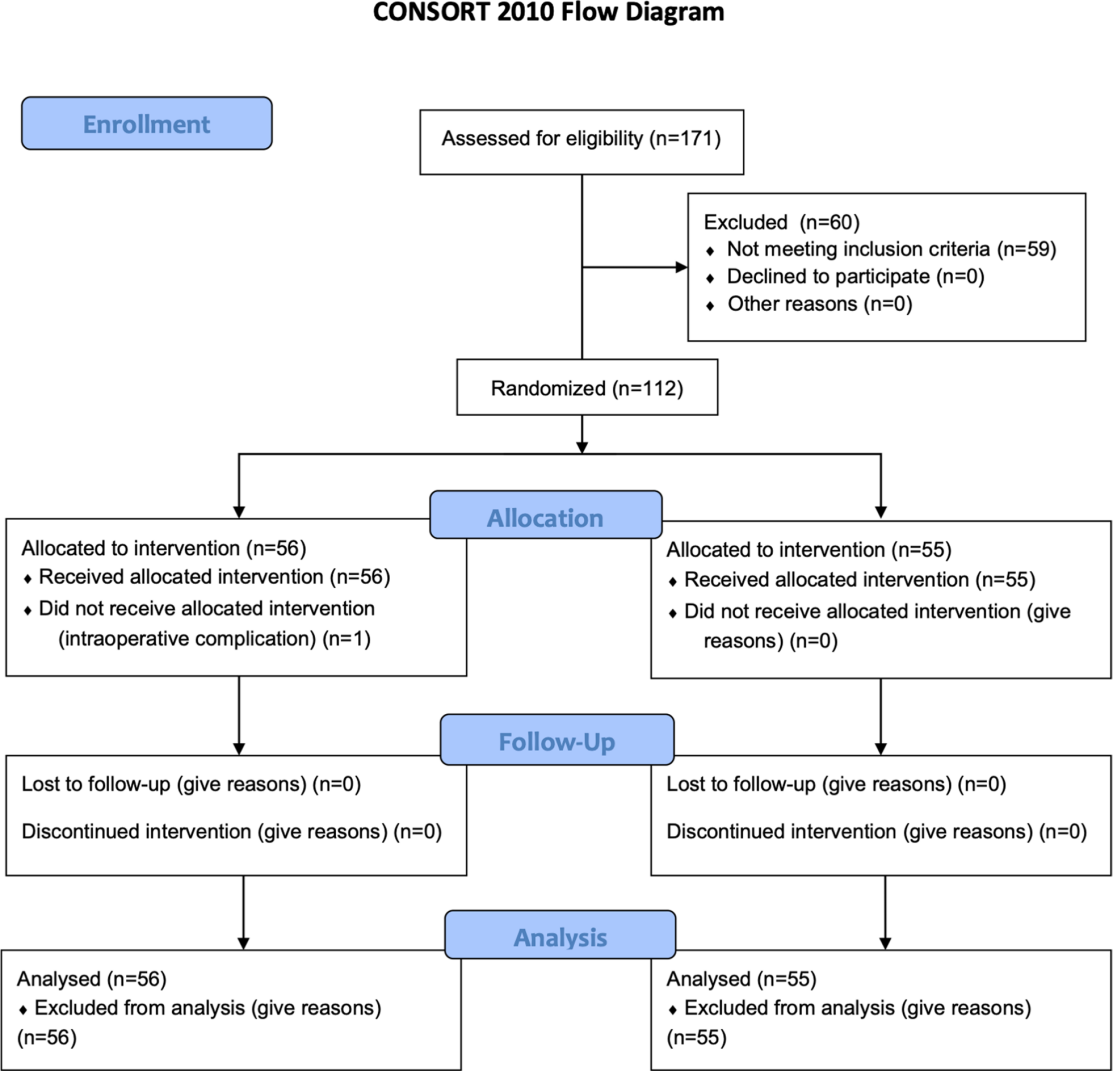
Study CONSORT checklist (Available at ) www.consort-statement.org

In this single-center, prospective, randomized, open label, phase-III study, consecutive patients with indication to ThuLEP for BPO were enrolled between December 1st, 2019 and February 28th, 2021 (ClinicalTrials.gov NCT05130918, registered on 23/11/2021). The study was approved by the local institutional ethics committee (no. approval STS N-726, Ethics Committee “Lazio 1”, “San Camillo Forlanini” Hospital, Rome, Italy) and performed in accordance with the ethical standards as laid down in the 1964 Declaration of Helsinki and its later amendments. Report of the trial conformed to the CONSORT 2010 guidelines (Fig. 1) [16]. The Landis criteria were acknowledged [17].

### Inclusion and exclusion criteria

Specifically for the purpose of the study, patients with history of prostatitis, history of neurogenic detrusor overactivity (as determined after urodynamic observation), diagnosed prostate cancer, previous surgeries of the lower urinary tract, indwelling catheter, history of bladder stones and/or nephrolithiasis, and known or suspected hypersensitivity to Phenolmicin P3 and/or Bosexil were excluded. Patients were excluded in case of preoperative positive urine culture or occurrence of severe intraoperative complications as well.

### Randomization

Eligible patients were randomized in a 1:1 ratio. The randomization scheme was generated by using the Web site Randomization.com (http://www.randomization.com). After randomization, the two arms were defined as follows: Group A: patients who were administered Phenolmicin P3 and Bosexil (Mictalase® medical device) suppositories twice a day for 5 days, then once a day for other 10 days as per the manufacturer's instructions; Group B: patients who did not receive Mictalase® (named “controls”).

### ThuLEP procedure

ThuLEP procedures were performed by three experienced surgeons (> 100 procedures performed before the study start) according to either a two-lobes or a three-lobes technique, depending on the anatomy of the adenoma, as previously described [18]. The procedure was performed using an Iglesias 26 F resectoscope, with a 4 mm, 12 degrees optics. The 200 Watt-Cyber-TM laser generator (Quanta System, Campagnano di Roma, Italy) was used with maximum power of 70 Watts set for cutting and 40 Watts set for coagulation. Low-power coagulation was used during almost the whole enucleation, while activating cutting in case of stickier tissue. A 550 nm laser fiber was used, with apical release performed at the beginning to reduce harms to the sphincter. The enucleated lobes were morcellated by the Piranha morcellator (Richard Wolf, Knittlingen, Germany).

### Outcomes measurements

Preoperative variables, including age, prostate volume (PVol), maximum urinary flow rate at uroflowmetry (Q_max_) with post-voiding residual volume (PVR), and International Prostate Symptom Score (IPSS) with Quality of Life (QoL) were collected. Patients were categorized into three clusters (mild: 0–11/moderate: 12–23/severe: 24–35 symptoms) based on IPSS score.

Perioperative data, including operative time, total laser energy delivered, enucleated adenoma grams, intraoperative complications classified according to the modified Satava system (Grade 1: complications included incidents without consequences for the patient; grade 2: complications which were treated intraoperatively with endoscopic surgery (grade 2a) or required endoscopic re-treatment (grade 2b); and grade 3: complications included incidents requiring open or laparoscopic surgery) [19], length of stay, and catheterization time were recorded. Eventual complications occurring within the early postoperative follow-up (30 days) were classified according to Clavien-Dindo [20].

Study endpoints were evaluated both at 15 and 30 days post-operative follow-up. Primary endpoint included the evaluation of the eventual effects of the suppository on irritative symptoms as assessed by the IPSS ad QoL questionnaires, with categorization of symptoms severity as aforementioned. Secondary endpoints included evaluation of the eventual effects of the suppository on the occurrence of urinary tract infections, assessed by performance of urinalysis with urine culture at the same time points (15 and 30 days post-operatively). Positive urine culture was reported as asymptomatic bacteriuria in patients without LUTS showing bacterial growth < 10^5^ CFU/ml on a mid-stream sample of urine [21]. In case of positive urine culture in patients with symptoms, appropriate antimicrobial therapy was prescribed based upon antibiogram.

Finally, although beyond the purpose of the study, to prove the adequate de-obstructive effect of ThuLEP, regardless of the allocation arm, patients underwent uroflowmetry with PVR estimation at 30-days follow-up.

### Sample size calculation

The target sample size for the primary outcome of interest was calculated assuming a 50% reduction of patients with moderate-to-severe symptoms (according to IPSS) after administration of the suppository under investigation.

Given a rate of patients with moderate-to-severe symptoms around 50% after ThuLEP (as per our previous experience), the rate was expected to drop to 25% at last follow-up in the group of patients administered with the suppository. With a 1-β power of 80% and a type I (α) error of 0.05, enrollment of 110 patients (55 per group) was required.

The sample size was adequate enough to evaluate eventual differences between the two groups in terms of postoperative alteration of the urine test at urinalysis or microscopic examination (red blood cells, white blood cells (or pus cells), bacteria (germs), and altered pH) and or positive urine culture (100% in our experience after ThuLEP versus 50% expected in patients administered with the suppository: 8 vs 8 patients to be enrolled).

### Statistical analysis

Continuous variables were summarized using medians and interquartile ranges (IQR); frequencies and proportions were used to report categorical variables. Median values of continuous variables calculated in the two study groups were compared by using the two-samples Mann Whitney test, while proportions of categorical variables were compared by using the Fisher's exact test.

Significance level was set at *p* value < 0.05. Statistical analysis was performed by using “Statistic” 8.0 Software (StatSoft, Tulsa, OK, United States).

## Results

A hundred-seventy-one consecutive patients were screened. Twenty-three patients with neurogenic detrusor overactivity, 5 with prostatitis, 10 with diagnosed prostate cancer, 3 who had undergone previous surgeries of the lower urinary tract, 2 with history of nephrolithiasis, and 16 with indwelling catheter were discarded. One patient who experienced trans-urethral resection syndrome (Satava 2a grade) was excluded from the analysis. No patient reported either known or suspected hypersensitivity to Phenolmicin P3 and/or Bosexil.

**Fig. 2:**
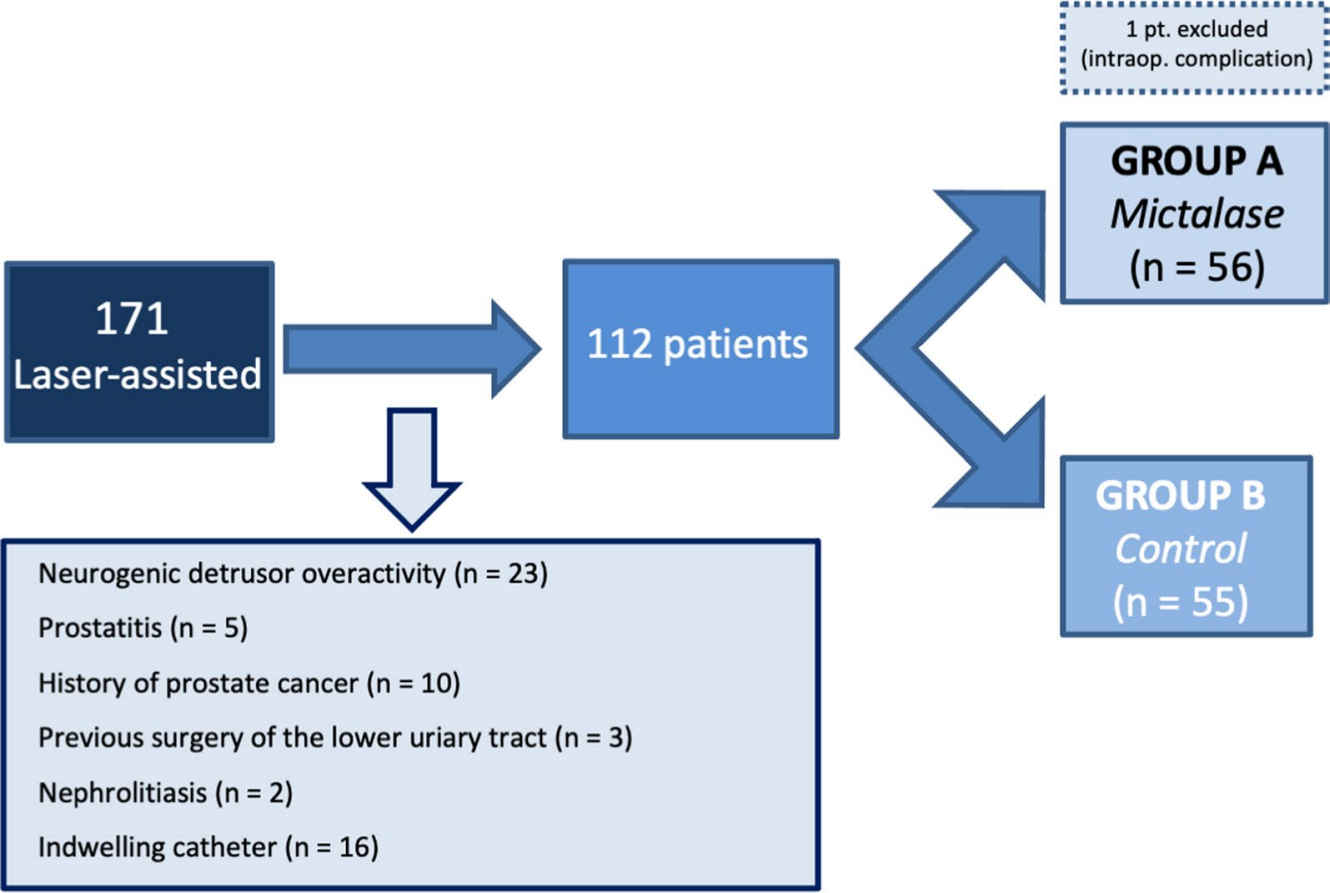
Study flow-chart

After accounting for the exclusion criteria, 111 randomized patients were analyzed. Fifty-six were allocated to treatment Group A and received Mictalase®; 55 were allocated to control Group B. The study flow-chart was reported in Fig. 2.

Groups were comparable at baseline in all variables analyzed. Concerning the intra-operative and peri-operative data, no statistically significant differences were found between the treatment groups.

Table 1 reported the complete data about baseline patients’ characteristics, intraoperative and peri-operative data stratified by treatment group.

**Table 1:** Distribution of baseline characteristics, peri-operative and post-operative outcomes of patients in the treatment groups

	Mictalase group (n = 56)	Control group (n = 55)	*p* value
*Patients’ baseline characteristics*			
Age, years	65 (61–75)	69 (63–74)	0.2
PVol, ml	73.5 (50.0–90.0)	70.0 (50.0–100.0)	0.7
Q_max_, ml/s	10.0 (8.0–13.7)	9.4 (7.0–12.1)	0.4
PVR, ml	65 (40–100)	90 (70–110)	0.5
IPSS	21 (16–26)	23 (19–28)	0.3
QoL	5 (4–5)	5 (4–6)	0.5
*Intra-operative data*			
Operative time, min	88 (70–125)	102 (75–138)	0.6
Energy delivered, joules	58 k (42–83 k)	60 k (44–80 k)	0.9
Enucleated adenoma, grams	63.5 (40–80)	60.0 (40–85)	0.7
*Peri-operative data*			
Length of stay, days	3 (2–3)	3 (3–4)	0.1
Catheterization time, days	3 (2–5)	3 (2–5)	0.1
Complications	2 (3.6)	3 (5.4)	0.6

Median is reported for continuous variables, while number of observations is reported for categorical variables. Inter-Quartile Range (IQR) and percentages are reported in brackets, as appropriate. PVol: Prostate Volume; Q_max_: maximum urinary flow rate at uroflowmetry; PVR: post-voiding residual volume; IPSS: International Prostate Symptom Score; QoL: Quality of Life

**Fig. 3:**
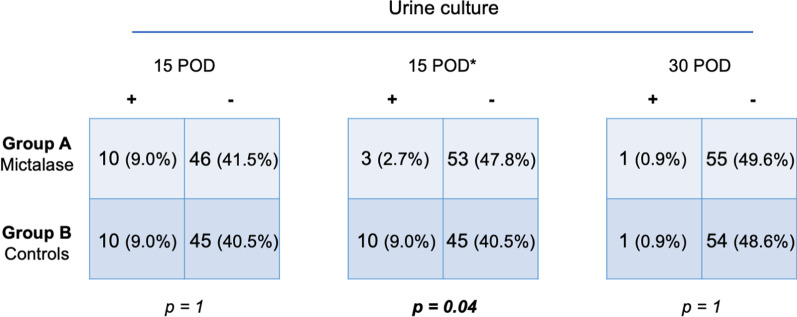
2 × 2 tables reporting urine culture data across treatment groups at 15th and 30th postoperative day (POD). *After excluding asymptomatic bacteriuria

At 15-days follow-up, no statistically significant differences were found comparing the treatment groups in terms of IPSS and QoL scores, and urinalysis parameters. Overall, rate of positive urine cultures was comparable, but after excluding asymptomatic bacteriuria, a statistically significant difference was found in favor of Group A (*p* = 0.04) (Table 2 and Fig. 3).

**Table 2:** Distribution of post-operative outcomes in the treatment groups

	Mictalase group (n = 56)	Control group (n = 55)	*p* value
*15th postoperative day follow-up*			
IPSS	12 (6–16)	10 (7–15)	0.7
QoL	2 (1–4)	2 (1–3)	0.1
Urine pH	6.5 (5.5–6.5)	5.5 (5.5–6.5)	0.4
WBC-sediment (counts/HPF)	90 (30–250)	55 (30–134)	0.4
RBC-sediment (counts/HPF)	90 (39–450)	66 (15–431)	0.5
Urine specific gravity	1013 (1008–1018)	1015 (1011–1020)	0.3
Positive urine culture	3 (2.7)	10 (9.0)	0.04
*30th postoperative day follow-up*			
IPSS	6 (3–11)	10 (5–13)	0.02
QoL	2 (1–3)	2 (1–4)	0.1
Urine pH	5.5 (5.5–7.0)	5.5 (5.0–6.0)	0.2
WBC-sediment (counts/HPF)	70 (11–138)	36 (25–81)	0.8
RBC-sediment (counts/HPF)	16 (7–65)	20 (8–113)	0.4
Urine specific gravity	1015 (1015–1019)	1014 (1010–1021)	0.9
Positive urine culture	1 (0.9)	1 (0.9)	1
Q_max_, ml/s	22.1 (19.0–28.2)	22.8 (16.4–25.2)	0.4
PVR, ml	0 (0–0)	0 (0–0)	0.6

Median is reported for continuous variables, while number of observations is reported for categorical variables. Inter-Quartile Range (IQR) and percentages are reported in brackets, as appropriate. IPSS: International Prostate Symptom score; QoL: Quality of Life; WBC: white blood cells; HPF: high power field; RBC: red blood cells, Q_max_: maximum urinary flow rate at uroflowmetry; PVR: post-voiding residual volume

When analyzing the patients stratified by clusters of IPSS score, 15th postoperative day follow-up showed comparable rates of patients with moderate-to-severe symptoms according to the IPSS score between the groups (27/56 (48.2%) versus 24/55 (43.6%), Group A versus Group B, respectively, *p* = 0.6).

**Fig. 4:**
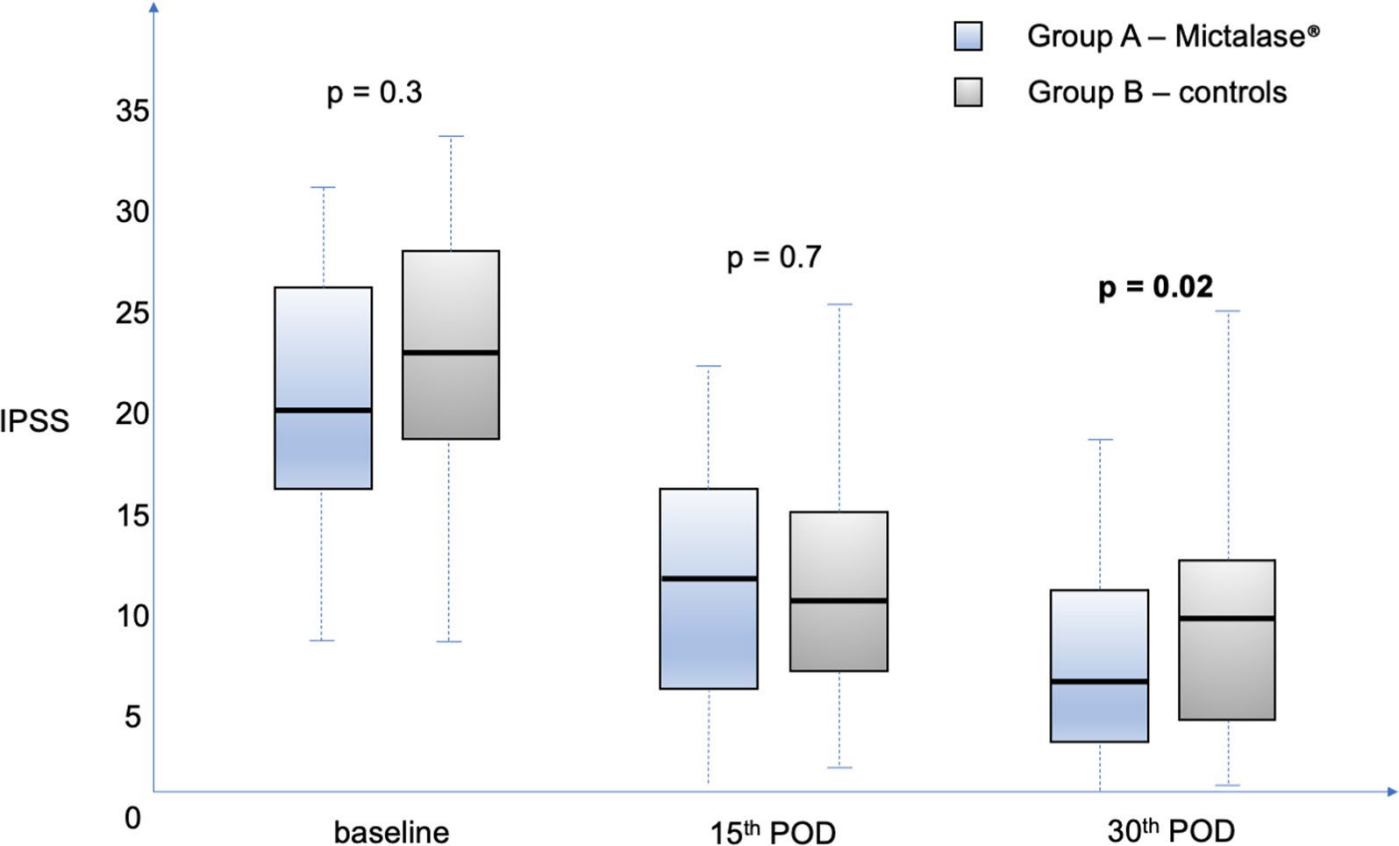
Box plot depicting the International Prostate Symptom Score (IPSS) in the groups (Group A—Mictalase® versus Group B—controls) at baseline, 15th and 30th postoperative day assessments

At 30-days follow-up, no significant differences were found in terms of QoL. Conversely, significant differences were found in the median IPSS score (6 [IQR 3–11] versus 10 [IQR 5–13], Group A vs B, respectively, *p* = 0.02). Figure 4 detailed the IPSS at different time points stratified by treatment groups. At reassessment of clusters of symptoms severity, 8 patients (14.3%) in Group A referred moderate symptoms (no patients had severe symptoms), whilst 19 patients (34.5%) in Group B still had moderate-to-severe symptoms (2/19 had severe symptoms) (*p* = 0.01). Urinalysis parameters and rate of positive urine cultures were not statistically significantly different (Table 2 and Fig. 3).

Finally, no statistically significant differences were found in Q_max_ and PVR (reported in Table 2).

## Discussion

The present single-center randomized controlled phase III trial investigated the efficacy of a suppository based on Phenolmicin P3 and Bosexil (Mictalase®) in control of irritative symptoms and prevention of lower urinary tract infections in patients undergoing ThuLEP.

After accounting for exclusion criteria, 111 patients were randomized (56 received Mictalase® versus 55 controls). Randomization performed well, with no differences at baseline between groups. Notably, although randomization would have been unable to control for intra-operative and peri-operative factors, no statistically significant differences were found between the groups. Moreover, ThuLEP performed well and similarly in relieving from BPO whatever the Group.

Concerning the study endpoints, improvement of IPSS at 30 days postoperation was more pronounced in patients who received Mictalase®. Moreover, a lower rate of positive urine culture at 15 days postoperatively favored the Mictalase® group.

To the best of our knowledge, no study investigated the use of Phenolmicin P3 and/or Bosexil, either alone or in combination to manage the post-operative irritative symptoms after transurethral prostate surgeries.

Even after expanding the search strings, to let the adoption of suppositories with different active principles being included, a few anecdotal studies are available in the field.

Within a prospective randomized controlled study, Ergakov et al. tested the efficacy of rectal suppository based on Serenoa repens, selenium and lycopene in association with antibiotics for prevention of infectious-inflammatory complications after TURP [22]. The authors found a statistically significant reduction in patient-reported outcomes (IPSS and QoL) in the treated group (11.5 ± 1.2 and 2.6 ± 0.3 points, respectively) compared with controls (15.5 ± 1.4 and 3.8 ± 0.5 points, respectively). Another study investigating the effects of the same medical device based on Serenoa repens, selenium and lycopene was published by Nozdrachev et al. who, by mean of inflammatory changes measured in postoperative blood and urine samples, and renal microcirculation including variations in perfusion intensity and renal ischemia and congestion, observed favorable response after administration of the suppository [23].

Other than the referenced experiences, no studies of interest were retrieved in our literature search. It is important to underline that the active principles included in the suppositories tested within the setting of the aforementioned studies (namely Serenoa repens, selenium and lycopene) are a well-known combination in the management of prostate-related symptoms. Furthermore, differently from the mentioned studies, we did not include the use of prolonged antibiotic prophylaxis in association with the investigated suppository.

The suppository we herein investigated includes different active principles, namely: Bosexil®, that is a vegetal extract derived from the resin of the *Boswellia serrata*, a plant native to India. It has already been published that the Boswellic acids contained show anti-inflammatory and antioxidant properties in a variety of inflammatory diseases whose physio-pathological pathways are shared with those of prostatitis [24, 25]; phenolmicin P3 is a polyphenolic extract derived from beehive propolis, that also demonstrated anti-inflammatory and antioxidant properties in preclinical reports. It has also been reported to have the ability to create a microenvironment hostile to the reproduction of pathogenic bacteria. Indeed, one of the most important etiological agents of inflammatory diseases is the cause-and-effect relationship between oxygen free radicals and oxidative damage at the biomolecular level [26–28]. Actually, the efficacy of the transrectal delivered association of Boswellia resin extract and propolis derived polyphenols in relieving prostatitis-like symptoms was tested by another research group [29]. As assessed by standardised questionnaires, the suppository was found able to reduce genitourinary pain and to improve quality of life in men affected by bothersome prostatitis-like symptoms.

In our randomized study, the suppository Mictalase® seemed to impact on the improvement in IPSS. The assessment of this patient-reported outcome after transurethral laser BPH surgery can be influenced by preoperative patient’s conditions (the baseline IPSS itself) and unmodifiable parameters (mostly PVol), intraoperative variables (mostly the total energy delivered), and postoperative factors (mostly the catheterization time and the occurrence of infections). Of note, even if most of the perioperative/postoperative variables would have remained uncontrolled by randomization, treatment groups did not significantly differ. Thus, it is hard to conclude that the differences we observed were due to chance. On the other hand, we underline the finding about two patients in the control group who remained with severe symptoms according to IPSS at the 30th day follow-up. These patients both had scored 34 at the baseline evaluation of IPSS, being “outliers” in the distribution of preoperative IPSS. This could have impacted on the persistence of severe symptoms after ThuLEP, mostly related to (compromised) bladder origin symptoms.

Another relevant finding from our study is the incidence of postoperative urinary infections as assessed by urine culture. Notwithstanding the known anti-microbial properties of phenolmicin P3, the exact mechanism of impact on the urine culture outcome is unclear. On the other hand, we observed that, in the setting of a randomized trial, clinically-significant urinary infections (requiring antibiotics) were anecdotal when Mictalase® was administered. This is interesting in the modern era, in which the abuse of antibiotic is discouraged by guidelines, due to epidemiological and socio-economics reasons [30]. The data we report herein would support the avoided routine use of antimicrobial prophylaxis beyond the perioperative single-shot even in the case of transurethral endoscopic procedure for BPH management [31].

Drafting conclusive recommendations on how to manage patients presenting with dysuria and/or pelvic pain and/or prostatodynia after transurethral prostate surgery still represents a challenge in endourology. Such syndrome remains of unclear pathophysiology, thus being a driver for stimulating further research. Moreover, the standardized assessment of the pain/discomfort relative to such syndrome still remains an open issue. For instance, in our study, we missed a dedicated evaluation of postoperative dysuria intended as painful/burning micturition felt either in the urethra or the perineum.

Although the rigorous methodology, given the number of actors playing a role in the complexity of post-transurethral prostate surgery syndrome, our study could have been underpowered in detecting other variables/effects. Moreover, the open label study design could have supersized the positive effect on irritative symptoms perceived in the treatment group on one hand; the absence of a “placebo” control could have worsened the outcomes measured in the control group on the other hand.

Last, we acknowledge the lack of any sort of blinding (either for patients or for investigators). This would have further improved the quality of the analysis.

More data about the actual impact of the combination of Phenolmicin P3 and Bosexil on the irritative symptoms and urinary infections in patients undergoing transurethral prostate surgery are warranted.

## Conclusions

The present randomized trial investigated for the first time the efficacy of the Mictalase® suppositories in the symptoms control and prevention of lower urinary tract infections in patients undergoing ThuLEP. Concerning the study endpoints, improvement of IPSS at 30 days postoperation was more pronounced in patients who received Mictalase®. Moreover, a lower rate of positive urine culture at 15 days postoperatively favored the Mictalase® group.

## Data Availability

The datasets generated and/or analyzed during the current study are not publicly available [due to their containing information that could compromise the privacy of research participants] but are available from the corresponding author on reasonable request.

## Abbreviations

LUTS: Lower urinary tract symptoms
BPH: Benign prostatic hyperplasia
BPO: Benign prostatic obstruction
TURP: Transurethral resection of the prostate
OP: Open prostatectomy
EEP: Endoscopic enucleation of the prostate
ThuLEP: Thulium laser enucleation of prostate
PVol: Prostate volume
Q_max_: Maximum urinary flow rate at uroflowmetry
PVR: Post-voiding residual volume
IPSS: International Prostate Symptom Score
QoL: Quality of Life
IQR: Interquartile ranges
